# Gender differences in the susceptibility of hospital-acquired acute kidney injury: more questions than answers

**DOI:** 10.1007/s11255-020-02526-7

**Published:** 2020-07-13

**Authors:** Helmut Schiffl

**Affiliations:** Department of Internal Medicine IV, University Hospital LMU Munich, Ziemssenstr. 1, 80336 Munich, Germany

**Keywords:** Hospital-acquired acute kidney injury, Sex dimorphism, Testosterone

## Abstract

Hospital-acquired acute kidney injury (HA-AKI) is a heterogeneous renal syndrome which occurs in different clinical settings. It is characterized by multiple aetiologies, various pathogeneses and unpredictable outcomes. HA-AKI, once predominantly viewed as a self-limited and reversible short-term condition, is now recognized as a harbinger for chronic kidney disease and a cause of long-term morbidity with an increased risk of cardiovascular, renal and cancer mortality. Recent clinical studies contradict the generally held belief that female sex is a risk factor for HA-AKI. They show, consistent with basic research performed with experimental models of AKI, that only male sex is associated with HA-AKI. The presence of testosterone, more likely than the absence of estrogen, plays a critical role in sex differences in the susceptibility of ischemia/reperfusion kidney injury. The conflicting data in epidemiological studies related to sex as susceptibility variable for human AKI, underscore the need for more rigorous, well designed observational studies taking into account the menopausal status and hormone therapy.

## Introduction

Hospital-acquired acute kidney injury (HA-AKI) is a complex clinical syndrome with multiple risk factors and aetiologies, a broad spectrum of clinical presentations and unpredictable short-and long-term outcomes. Currently, treatment of established acute kidney injury (AKI) is mainly supportive in nature. No pharmacological intervention has shown efficacy to improve morbidity and mortality attributable to AKI. Identifying patients at high risk, awareness of preventable AKI, early recognition and management of incipient AKI are key factors to reduce cases of established AKI and to improve the dismal outcome of severe AKI [1].

Observational studies have identified a variety of risk factors including pre-existing susceptibilities of individual patients in combination with the nature and severity of acute kidney insults. Sex differences are of fundamental importance in chronic kidney disease (CKD). Biological sex is increasingly recognized as modulator of the pathophysiology, disease development, progression and management of CKD. Male patients with proteinuric CKD have a faster progression of CKD to end-stage kidney disease (ESKD) than women with the same grade of proteinuria, indicating that female sex may be reno-protective [2]. Sexual dimorphism has also been examined in HA-AKI. However, observational studies in humans reported conflicting findings [3] such as: (1) women have a greater risk for HA-AKI; (2) no association between sex and HA-AKI was found in subpopulations; (3) women exhibit a decreased susceptibility to HA-AKI.

This narrative review starts with a critical appraisal of published studies, and thereafter discusses the proposed sex hormone effects for the pathogenesis of HA-AKI and for the transition of AKI to CKD.

### Sex and HA-AKI

At present, there are no randomized controlled trials examining sex as a susceptibility variable for HA-AKI. Most clinical cohort studies are confounded by the inclusion of women across all age groups including pre- and post-menopausal women in an uncontrolled way.

Older epidemiologic studies focused on male-only or male-predominant populations claimed that female sex was associated with higher risk for AKI after cardiac surgery, contrast -induced nephropathy or drug-induced nephrotoxicity. Consequently, female sex has been included as a risk factor in models developed to predict the risk of these AKI aetiologies [4]. The KDIGO (Kidney Disease Improving Global Outcomes) Practice guidelines stated that contrary to most CKDs, female sex was among the shared susceptibility factors that confer a high risk of HA-AKI [5]. Confounding systematic bias and insufficient statistical power in these studies has resulted in the wrong assumption that female sex may enhance susceptibility for HA-AKI. However, it was generally assumed, that data obtained from research involving male subjects could be simply extrapolated to women, and therefore, there was apparently no need to include an adequate number of female subjects in clinical studies.

With the advent of guidance documents and USA federal law (1993 National Institute of Health Revitalization Act) for enrolment of women in clinical research, a new awareness of the distinction between women and men evolved. Recently, Neugarten et al. analysed 28 studies from 2003 to 2018 (6,758,124 patients; 2,313,202 women and 4,444,922 men) that utilized multivariate analysis to asses risk factors for various aetiologies of HA-AKI and provided sex-stratified odds ratios. The results of this meta-analysis demonstrated that female sex is associated with protection against AKI and undermined the previously established belief that female sex is a significant risk factor for AKI. On the contrary male sex seems to enhance the susceptibility for HA-AKI. Subgroup meta-analyses showed that male sex was associated with an increased risk in unselected hospitalised patients, in critically ill patients, and in patients undergoing non-cardiac surgery. There was no association of sexual dimorphisms and post-cardiac surgery AKI and contrast-induced nephropathy [6].

However, residual measured, and non-measured confounding persists when using large health data bases. Biases may influence not only the result of a well performed observational study but also the conclusions made by the authors of meta-analyses.

Existing big data of hospitalized patients may be incomplete, inaccurate or inconsistently measured, and may explain the failure to show an association of sexual dimorphism and specific aetiologies of HA-AKI.

The diagnosis of HA-AKI is currently guided by changes in serum creatinine, urine output, or both over time [5]. Thus, a major limitation of meta-analyses relates to the inherent difficulty to identify HA-AKI in men relative to women in the light of sex-related differences in creatinine kinetics. Current classification systems for AKI differ regarding the increases in serum creatinine levels and in the observation time necessary to detect threshold changes in serum creatinine levels. The sex-stratified incidence of HA-AKI also varies according to the classification system used [7]. Srisawat et al. showed that the incidence of HA-AKI was greater in men than women when KDIGO criteria were used to define AKI, but that sex-related differences in HA-AKI disappeared when RIFLE criteria were used [8]. Consistent with these observations, the subgroup analysis by Neugarten et al. showed, that female sex was more likely associated with protection from AKI in those studies which utilized KDIGO criteria than in those that used RIFLE criteria [6].

Observational studies repeatedly demonstrated in unadjusted analyses, that the incidence of contrast induced nephropathy (CIN) was greater in women than in men. However, procedural factors such as the total volume of contrast media or the type and osmolarity of contrast media are crucial for the development of CIN. The association of CIN incidence and female sex may merely reflect a higher dose of contrast media administered to women than to men. Women generally have lower body weights or body surface areas than men, and accordingly the volume of contrast media administered, when expressed as total volume or as volume per body surface area has frequently been reported to be higher in women than in men [9]. Numerous studies utilizing multivariate analysis found that the variable “sex” is not associated with CIN.

Different patient populations may be characterized by different risk factors that convey different levels of risk for HA-AKI [10, 11]. Postcardiac surgery AKI relates to multiple patient-, acuity of the disease-, and surgical procedure-related risk factors. Women undergoing cardiac surgery often have more comorbid conditions which may mask the female sex-related protection of HA-AKI [12].

A small cohort study with 35 patients fulfilling the strict diagnostic criteria for the systemic inflammatory response syndrome (SIRS), found that pre-menopausal women seem to be protected from both AKI and acute respiratory distress syndrome (ARDS) [13]. It has been demonstrated, that the lower susceptibility to HA-AKI in women may be lost in aged women [14, 15].

### Sex and recovery of kidney function from HA-AKI

Patients who survive HA-AKI have an increased risk for progressive CKD, depending on the nature, severity and duration of the acute renal dysfunction and the presence of pre-existing CKD. Weinhandl et al. reported at the 2019 Kidney week conference of the American Society of Nephrology, that younger age, female sex and lower serum creatinine were among the demographic and biochemical variables most strongly associated with recovery of kidney function after initiation of outpatient haemodialysis for AKI in 12,221 patients [16]. Female sex was significantly associated with a 24% increased likelihood of recovering kidney function and a 14% decreased likelihood of transitioning to ESKD.

### Sex and susceptibility of rodents to experimental AKI models

Experimental science is producing tantalizing clues that sex differences influence the susceptibility to AKI and the recovery of renal function from AKI. Rodents (rats, mice) are genetically similar to humans, and are often used for experimental models of AKI. These animal studies have repeatedly suggested that sexual hormones (testosterone, testosterone/estrogen ratio, estrogen) are important determinants of sex differences in AKI susceptibility [17–23]. Female rodents (mice, rats) are much more resistant to ischemic renal injury when compared with male rodents. This protective effect of female sex has been consistently observed in models of ischemia–reperfusion (IR) injury, cardiac arrest/cardiopulmonary resuscitation and endoplasmic reticulum stress-induced kidney injury. Park et al. demonstrated that presence of testosterone, more than the absence of estrogen, induces kidney injury associated with IR [23]. Testosterone administration to female mice increased the kidney susceptibility to ischemia. Castration reduced IR induced kidney injury. In contrast, oophorectomy did not affect kidney injury induced by ischemia in female mice. Patil et al. tested the hypothesis that different levels of testosterone promote renal IR [24]. Their data suggested that the in contrast to acute high doses of testosterone, acute low dose testosterone was protective against IR AKI in male rats due to its effects on inflammation by reducing renal T cell infiltration and by shifting the balance in favour of anti-inflammatory cytokines.

Experiments involving rats found that there was a sexual dimorphism in the post-ischemic AKI to CKD transition and the reno-protection observed with female rats was lost with oophorectomy. The authors concluded that female sex hormones were responsible for the reno-protection observed. Early antioxidant defence and higher TGFβ, HIF1α and eNOS were among the reno-protective mechanisms that were demonstrated in female rats after IR [21].

Recent advances have led to a greater appreciation of how mitochondrial dysfunction contributes to the pathogenesis of ischemic or septic AKI, from decreased ATP production, to enhanced mitochondrial oxidative stress, cell necrosis and apoptosis. There are important sex hormone-related differences in mitochondrial respiration, biogenesis and dynamics [22].

However, it is important to be cautious when interpreting pathophysiologic findings and therapeutic interventions derived from the existing animal models of AKI. The development of animal models that manifest comorbid factors commonly known to affect the immune response and increase the risk of AKI is imperative to increase the clinical applicability of such observations. Current models of septic AKI do not reproducibly result in kidney injury. The absence of sepsis-related comorbidities is also a limiting factor that needs to be considered when translating findings derived from experimental AKI models to humans.

**Fig. 1:**
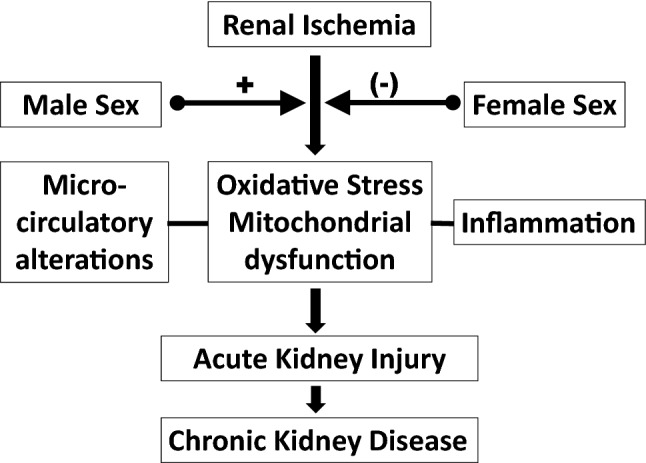
Putative mediators of the impact of sex on acute kidney disease

Finally, little is known about the cellular and molecular mechanisms underlying these sex differences in experimental models of AKI (Fig. 1).

## Conclusions

Experimental models of AKI clearly show a deleterious effect of testosterone and a protective effect of estrogen, but the data in humans are less clear. The conflicting epidemiologic data underscore the need for more rigorous, well designed, observational studies. These studies should adjust for the menopausal status and the history of hormone therapy.
